# Supplementary material for: Smooth Interpolating Curves with Local Control and Monotone Alternating Curvature
Source: Comput Graph Forum. 2022 Oct 6;41(5):25–38. doi: 10.1111/cgf.14600 (PMC9827861; doi:10.1111/cgf.14600)
Supplement: Supplementary file 1 — Supplement Material [file CGF-41-25-s001.zip › Local-Smooth-Interpolating-MonoCurvature/extern/clothoids/submodules/quarticRootsFlocke/docs/api-cpp/program_listing_file_PolynomialRoots-Utils.cc.html]

Program Listing for File PolynomialRoots-Utils.cc — Quartic Roots v1.0.0

### Navigation

- index
- toc
- Quartic Roots »
- Program Listing for File PolynomialRoots-Utils.cc

# Program Listing for File PolynomialRoots-Utils.cc¶

↰ Return to documentation for file (`PolynomialRoots-Utils.cc`)

```
/*--------------------------------------------------------------------------*\
 |                                                                          |
 |  Copyright (C) 2017                                                      |
 |                                                                          |
 |         , __                 , __                                        |
 |        /|/  \               /|/  \                                       |
 |         | __/ _   ,_         | __/ _   ,_                                |
 |         |   \|/  /  |  |   | |   \|/  /  |  |   |                        |
 |         |(__/|__/   |_/ \_/|/|(__/|__/   |_/ \_/|/                       |
 |                           /|                   /|                        |
 |                           \|                   \|                        |
 |                                                                          |
 |      Enrico Bertolazzi                                                   |
 |      Dipartimento di Ingegneria Industriale                              |
 |      Universita` degli Studi di Trento                                   |
 |      email: enrico.bertolazzi@unitn.it                                   |
 |                                                                          |
\*--------------------------------------------------------------------------*/

#ifdef __GNUC__
#pragma GCC diagnostic ignored "-Wunused-function"
#endif
#ifdef __clang__
#pragma clang diagnostic ignored "-Wglobal-constructors"
#pragma clang diagnostic ignored "-Wvla-extension"
#pragma clang diagnostic ignored "-Wvla"
#pragma clang diagnostic ignored "-Wunused-function"
#pragma clang diagnostic ignored "-Wdocumentation-unknown-command"
#endif

#include "PolynomialRoots.hh"
#include "PolynomialRoots-Utils.hh"

#ifndef DOXYGEN_SHOULD_SKIP_THIS

namespace PolynomialRoots {

  // static valueType const machepsi = std::numeric_limits<valueType>::epsilon();

  using std::pair;
  using std::abs;
  using std::pow;

  // - - - - - - - - - - - - - - - - - - - - - - - - - - - - - - - - - - - - - -
  // stable computation of polinomial
  //
  // op[0] * x^n + .... + op[n-1]*x + op[n]
  //
  // (op[0] + op[1]/x.... + op[n]/x^n)*x^n
  //
  valueType
  evalPoly(
    valueType const op[],
    indexType       Degree,
    valueType       x
  ) {
    bool reverse = std::abs(x) > 1;
    if ( reverse ) {
      valueType res(op[Degree]);
      valueType xn(1);
      for ( indexType i = 1; i <= Degree; ++i ) {
        res = res/x + op[Degree-i];
        xn *= x;
      }
      res *= xn;
      return res;
    } else {
      valueType res(op[0]);
      for ( indexType i = 1; i <= Degree; ++i ) res = res*x + op[i];
      return res;
    }
  }

  // - - - - - - - - - - - - - - - - - - - - - - - - - - - - - - - - - - - - - -
  // stable computation of Newton step
  //
  // op[0] * x^n + .... + op[n-1]*x + op[n]
  //
  // (op[0] + op[1]/x.... + op[n]/x^n)*x^n
  //
  bool
  NewtonStep(
    valueType const op[],
    indexType       Degree,
    valueType     & x
  ) {
    valueType p, dp;
    bool reverse = std::abs(x) > 1;
    if ( reverse ) {
      p = op[Degree];
      valueType xn(1);
      for ( indexType i = 1; i <= Degree; ++i ) {
        p = p/x + op[Degree-i];
        xn *= x;
      }
      p *= xn;

      dp = op[Degree];
      xn = 1;
      for ( indexType i = 2; i <= Degree; ++i ) {
        dp = dp/x + (Degree-i)*op[Degree-i];
        xn *= x;
      }
      dp *= xn;
    } else {
      dp = Degree*op[0];
      p  = op[0];
      for ( indexType i = 1; i < Degree; ++i ) {
        p  = p*x  + op[i];
        dp = dp*x + (Degree-i)*op[i];
      }
      p = p*x + op[Degree];
    }
    x -= p/dp;
    return true;
  }

  // - - - - - - - - - - - - - - - - - - - - - - - - - - - - - - - - - - - - - -
  // stable computation of polinomial and its derivative
  //
  // op[0] * x^n + .... + op[n-1]*x + op[n]
  //
  // (op[0] + op[1]/x.... + op[n]/x^n)*x^n
  //
  void
  evalPolyDPoly(
    valueType const op[],
    indexType       Degree,
    valueType       x,
    valueType     & p,
    valueType     & dp
  ) {
    bool reverse = std::abs(x) > 1;
    if ( reverse ) {
      valueType const * pc = op+Degree;
      valueType rD = Degree;
      p  = *pc--;
      dp = rD*p;
      valueType xn(1);
      for ( indexType i = 1; i < Degree; ++i ) {
        p  = p/x  + *pc;
        dp = dp/x + rD*(*pc--);
        xn *= x;
      }
      dp *= xn;
      p   = p/x + *pc;
      xn *= x;
      p  *= xn;
    } else {
      p = op[0]*x+op[1];
      for ( indexType i = 2; i <= Degree; ++i ) {
        p  = p*x  + op[i];
        dp = dp*x + i*op[i];
      }
    }
  }

  // - - - - - - - - - - - - - - - - - - - - - - - - - - - - - - - - - - - - - -
  //
  // op[0] * x^n + .... + op[n-1]*x + op[n]
  std::complex<valueType>
  evalPolyC(
    valueType const                 op[],
    indexType                       Degree,
    std::complex<valueType> const & x
  ) {
    bool reverse = std::abs(x) > 1;
    if ( reverse ) {
      std::complex<valueType> res(op[Degree]);
      std::complex<valueType> xn(1,0);
      for ( indexType i = 1; i <= Degree; ++i ) {
        res = res/x + op[Degree-i];
        xn *= x;
      }
      res *= xn;
      return res;
    } else {
      std::complex<valueType> res(op[0]);
      for ( indexType i = 1; i <= Degree; ++i ) res = res*x + op[i];
      return res;
    }
  }

  //============================================================================

  /*
  ..  scale roots by pair.second, after scaling the polinomial has the form
  ..
  ..  p[0] + p[1]*x + ... + p[n]*x^n
  ..
  ..  pair.first is the index such that p[pair.first] = +-1
  ..
  */

  static
  pair<indexType,valueType>
  scalePolynomial(
    indexType       n, // degree
    valueType const p[],
    valueType       ps[]
  ) {
    indexType i_max = n;
    valueType an    = p[n];
    valueType scale = -1;
    indexType i = n;
    while ( --i >= 0 ) {
      ps[i] = p[i]/an;
      valueType scale1 = std::pow( std::abs(ps[i]), 1.0/(n-i) );
      if ( scale1 > scale ) { scale = scale1; i_max = i; }
    }
    // scale coeffs
    pair<indexType,valueType> res(i_max,scale);
    valueType s = scale;
    for ( i = 1; i <= n; ++i, s *= scale ) ps[n-i] /= s;
    ps[n] = 1;
    return res;
  }

  //============================================================================

  /*
  .. divide a(x)  by (x-r) so that a(x) = (x-r) * b(x)
  */

  static
  void
  deflatePolynomial(
    indexType       n, // degree
    valueType const a[],
    valueType       r,
    valueType       b[]
  ) {
    // crossover index for forward/backward deflation
    // G. Peters and J. H. Wilkinson.
    // Practical problems arising in the solution of polynomial equations.
    // J. Inst. Math. Appl. 8 (1971), 16–35.
    indexType i_cross = 0;
    valueType v_cross = std::abs(a[0]);
    valueType r1 = r;
    for ( indexType i = 1; i < n; ++i, r1 *= r ) {
      valueType v_cross1 = std::abs(a[i]*r1);
      if ( v_cross1 > v_cross )
        { v_cross = v_cross1; i_cross = i; }
    }
    b[n-1] = 1;
    if ( isZero(a[n]-1) ) {
      if ( i_cross > 0 ) {
        b[0] = -a[0] / r;
        for ( indexType j = 1; j < i_cross; ++j )
          b[j] = (a[j]-b[j-1]) / r;
      }
      for ( indexType j = n-2; j >= i_cross; --j )
        b[j] = a[j+1]+r*b[j+1];
    } else {
      valueType an = a[n];
      if ( i_cross > 0 ) {
        b[0] = -(a[0]/an) / r;
        for ( indexType j = 1; j < i_cross; ++j )
          b[j] = (a[j]/an-b[j-1]) / r;
      }
      for ( indexType j = n-2; j >= i_cross; --j )
        b[j] = a[j+1]/an+r*b[j+1];
    }
  }

  // - - - - - - - - - - - - - - - - - - - - - - - - - - - - - - - - - - - - - -
  // stable computation of polinomial
  // p0 + p1*x + p2*x^2 + ... + pn*x^n
  //
  // (p0/x^n + p1/x^(n-1) + p2/(x^(n-2) + ... + pn)*x^n
  //
  #if 0
  // UNUSED
  valueType
  CompHorner(
    valueType const p[],
    indexType       Degree,
    valueType       x
  ) {
    valueType xabs = std::abs(x);
    bool reverse = xabs > 1;
    //bool reverse = false;
    if ( reverse ) x = valueType(1)/x;
    indexType ii0 = reverse ? Degree : 0;
    valueType res(p[ii0]);
    valueType c = 0;
    for ( indexType i = 1; i <= Degree; ++i ) {
      indexType ii = reverse ? Degree-i : i;
      valueType tmp, pi, sigma;
      //TwoProduct( res, x, tmp, pi );
      //TwoSum( tmp, p[ii], res, sigma );
      res = res * x + p[ii];
      //c = c * x + (pi+sigma);
    }
    //res += c;
    if ( reverse ) res *= std::pow(x,Degree);
    return res;
  }
  #endif

  // - - - - - - - - - - - - - - - - - - - - - - - - - - - - - - - - - - - - - -
  //
  // x^3 + A x^2 + B x + C
  static
  inline
  void
  scaleCubicMonicPolynomial(
    valueType   A,
    valueType   B,
    valueType   C,
    valueType & AS,
    valueType & BS,
    valueType & CS,
    indexType & i_case,
    valueType & scale
  ) {

    valueType a = std::abs(A);
    valueType b = std::sqrt(abs(B));
    valueType c = std::cbrt(abs(C));

    if ( a < b ) {
      if ( b < c ) i_case = 0; // a < b < c --> c MAX
      else         i_case = 1; // a < b and c <= b --> b MAX
    } else {
      if ( a < c ) i_case = 0; // b <= a < c --> c MAX
      else         i_case = 2; // b <= a  and c <= a --> a MAX
    }

    switch ( i_case ) {
    case 0:
      scale = c;
      AS    = A/c;
      BS    = (B/c)/c;
      CS    = C > 0 ? 1 : -1;
      break;
    case 1:
      scale = b;
      AS    = A/b;
      BS    = B > 0 ? 1 : -1;
      CS    = ((C/b)/b)/b;
      break;
    case 2:
      scale = a;
      AS    = A > 0 ? 1 : -1;
      BS    = (B/a)/a;
      CS    = ((C/a)/a)/a;
      break;
    }
  }

  // - - - - - - - - - - - - - - - - - - - - - - - - - - - - - - - - - - - - - -
  //
  // a3*x^3 + a2*x^2 + a1*x + a0 = (x-r)*(a3*x^2+b1*x+b0)
  static
  void
  deflateCubicPolynomial(
    valueType   a3,
    valueType   a2,
    valueType   a1,
    valueType   a0,
    valueType   r,
    valueType & b1,
    valueType & b0
  ) {
    indexType i_cross  = 0;
    valueType r2       = r*r;
    valueType v_cross  = std::abs(a0);
    valueType v_cross1 = std::abs(a1*r);
    if ( v_cross1 > v_cross ) { v_cross = v_cross1; i_cross = 1; }
      v_cross1 = std::abs(a2*r2);
      if ( v_cross1 > v_cross ) { v_cross = v_cross1; i_cross = 2; }
      v_cross1 = std::abs(a3*r*r2);
      if ( v_cross1 > v_cross ) i_cross = 3;
      switch ( i_cross ) {
      case 0: b1 = a2+a3*r; b0 = a1+r*b1; break;
      case 1: b1 = a2+a3*r; b0 = -a0/r;   break;
      case 2:
      case 3: b0 = -a0/r; b1 = (b0-a1)/r; break;
    }
  }

}

#endif

// EOF: PolynomialRoots-Utils.cc
```

### Quick search

### Table of Contents

- Quartic Roots Solver
- C++ API

«
hide menu

menu
sidebar
»

### Navigation

- index
- toc
- Quartic Roots »
- Program Listing for File PolynomialRoots-Utils.cc

© Copyright 2021, Enrico Bertolazzi.
Created using Sphinx 4.2.0.
